# Microbial electrosynthesis: is it sustainable for bioproduction of acetic acid?†

**DOI:** 10.1039/d1ra00920f

**Published:** 2021-03-08

**Authors:** Siddharth Gadkari, Behzad Haji Mirza Beigi, Nabin Aryal, Jhuma Sadhukhan

**Affiliations:** Department of Chemical and Process Engineering, University of Surrey Guildford GU2 7XH UK s.gadkari@surrey.ac.uk; Centre for Environment and Sustainability, University of Surrey Guildford Surrey GU2 7XH UK; Department of Microsystems, University of South-Eastern Norway Horten Norway

## Abstract

Microbial electrosynthesis (MES) is an innovative technology for electricity driven microbial reduction of carbon dioxide (CO_2_) to useful multi-carbon compounds. This study assesses the cradle-to-gate environmental burdens associated with acetic acid (AA) production *via* MES using graphene functionalized carbon felt cathode. The analysis shows that, though the environmental impact for the production of the functionalized cathode is substantially higher when compared to carbon felt with no modification, the improved productivity of the process helps in reducing the overall impact. It is also shown that, while energy used for extraction of AA is the key environmental hotspot, ion-exchange membrane and reactor medium (catholyte & anolyte) are other important contributors. A sensitivity analysis, describing four different scenarios, considering either continuous or fed-batch operation, is also described. Results show that even if MES productivity can be theoretically increased to match the highest space time yield reported for acetogenic bacteria in a continuous gas fermenter (148 g L^−1^ d^−1^), the environmental impact of AA produced using MES systems would still be significantly higher than that produced using a fossil-based process. Use of fed-batch operation and renewable (solar) energy sources do help in reducing the impact, however, the low production rates and overall high energy requirement makes large-scale implementation of such systems impractical. The analysis suggests a minimum threshold production rate of 4100 g m^−2^ d^−1^, that needs to be achieved, before MES could be seen as a sustainable alternative to fossil-based AA production.

## Abstract 2

Detailed LCA study of acetic acid production using microbial electrosynthesis to explore scenarios when this process could become sustainable.

## Graphical abstract

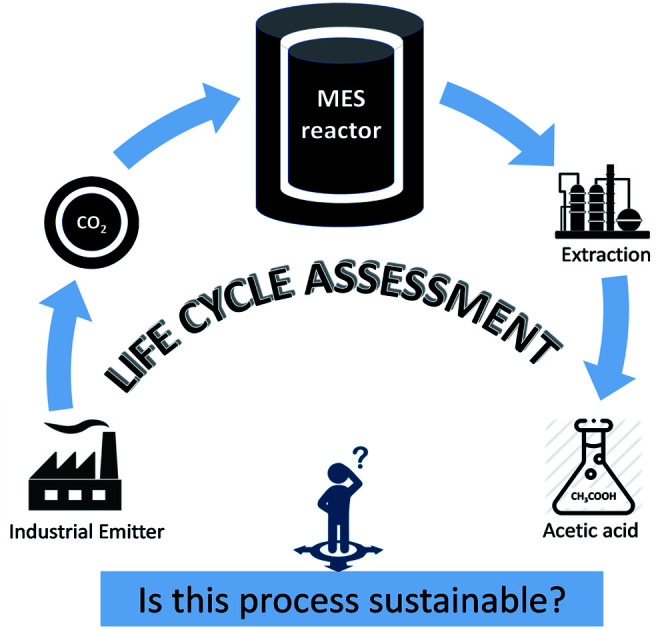

## Introduction

Depleting natural resources, threat of climate change and continuous increase in demand for chemicals and fuels (due to rising living standards and growing world population), have led to a surge in research for technologies that can provide sustainable alternatives to traditional fossil-based routes of chemical commodities production. Microbial electrosynthesis (MES), based on bioelectrochemical systems (BESs), offers one such potentially sustainable route of producing useful platform and commodity chemicals.^1,2^ MES does not involve the use of fossil-based substrates, but instead uses microorganisms' metabolism to reduce CO_2_ to valuable chemicals.^3,4^ Versatility of the bacterial culture (microbiome) that can be used as biocatalyst in MES allows it to be used for bioproduction of a wide range of chemicals such as different acids (formic, acetic, caproic, butyric, succinic, *etc.*), alcohols (methanol, ethanol, butanol, isopropanol, *etc.*), and fuels (CH_4_, H_2_), among others.^5–7^ Out of these, the most common end-product and the one which has showed the highest production rate so far, is acetic acid (AA).

AA is an important commodity chemical and is employed in the production of a number of useful chemicals such as vinyl acetate monomer, ethyl acetate, polyethylene terephthalate (PET), cellulose acetate, *etc.*, either as a raw material or an intermediate.^8^ These chemicals have applications in wide range of industries such as textile, food, automobile and construction. Growing requirement from these industries is leading the growth in acetic acid demand, which was 12.1 million tons in 2014 and is projected to increase to 16.8 million tons by 2022.^9^ Global market for AA is forecasted to reach value of $11.4 billion by 2024.^10^ Fossil-based AA production has high carbon footprint, and therefore alternative sustainable approaches are being pursued.^11^

MES along with some other biomass-based bioconversion technologies have shown promising results.^4,12–14^ From its first demonstration in 2009–10, performance of MES has improved many-fold.^3,6^ This is evident from the fact that concentration of microbially electrosynthesized AA has increased from 0.6 g L^−1^ to 12.4 g L^−1^ in the past decade. Along with product titer, area-specific AA production rates have also increased from 1.3 g m^−2^ d^−1^, all the way up to 685 g m^−2^ d^−1^.^6,15,16^ However, when compared to traditional routes of production, yield and final product concentration from MES are still very low and need to be improved substantially before this technology can be scaled-up.^6^

Among the different approaches that are being investigated, development of new highly efficient porous cathodes that offer high surface area, enhanced microbe-electrode interaction, screening of new electroactive species, and substrate diffusion, have played a major role in improving MES performance.^17–20^ While it is imperative to pursue all avenues that can help in enhancing MES productivity, it is also critical to evaluate the environmental impact of the process to determine the overall sustainability.^2,21^

Life cycle assessment (LCA) is an effective and standard methodology used to determine the environmental impact of a process.^22^ So far very few studies have investigated the sustainability of BESs, and among these, only a couple have focussed on MES. Foley *et al.*^23^ conducted LCA for large scale microbial fuel cell (MFC, used for electricity generation) and microbial electrolysis cell (MEC, used for hydrogen peroxide production) by assuming high current density (1000 A m^−3^) and compared them with the anaerobic treatment technology conventionally adopted for medium-strength industrial wastewater. It was shown that even with the presumed high current density, MFC reactor did not provide any major advantage compared to the conventional treatment method, however MEC, on account of chemical production, did show environmental benefits. Pant *et al.*^24^ demonstrated the importance of LCA of BESs and provided general recommendations on the appropriate functional unit and system boundary definitions that would be useful in such analyses. Zhang *et al.*^25^ developed an LCA model for microbial desalination cell (MDC) and studied the environmental impacts during system manufacturing, pre-treatment and operation. The model predicted that use of alternative materials in construction, and improvement in power density could help improve the sustainability of MDCs in future.

Christodoulou *et al.*^26^ performed a cradle-to-gate LCA analysis of MES, targeting the production of five different chemicals; acetic acid, formic acid, ethanol, propionic acid and methanol. The sustainability assessment was based on three impact indicators; net energy consumption, energy gain and global warming ratio. While the energy values for acetic and formic acid were adopted from experiments, the final product concentrations were assumed in the model. For the case of AA, Christodoulou *et al.*^26^ assumed high product titer of 41.3 wt%. Impact assessment showed that a large portion of total energy was consumed in product separation and purification, closely followed by energy consumed in the reactor operation. For the system studied by Christodoulou *et al.*,^26^ all three impact indicators suggested that AA production using MES was not sustainable in comparison to conventional petrochemical route. It should be noted that Christodoulou *et al.*^26^'s analysis is based on energy data for just one electrode, cathode. Okoroafor *et al.*^27^ expanded Christodoulou *et al.*^26^'s work and included the energy load for both anode and cathode. Okoroafor *et al.*^27^ obtained similar results and showed negative environmental impact in all impact categories for AA. They further compared MES with three other production methods but limited their study to formic acid.

Neither Christodoulou *et al.*^26^ nor Okoroafor *et al.*^27^ have discussed the contributions of individual components of the MES reactor. The reactor impact is calculated as a whole. Information about individual components and sub-processes, if available, can help to focus optimization efforts and improve process sustainability. Also, the final product titer in these studies is arbitrary and a high value has been assumed without justification.

The objective of this study is to address these issues and assess the sustainability of bioproduction of AA *via* MES. Towards this goal, a detailed contribution analysis, identifying the environmental hotspots of the production process is presented. Considering that large section of MES research is focussed on AA production, it is important to establish if such great efforts have helped the technology to move towards sustainable production. Future scenarios are also explored in a sensitivity analysis with different sources of energy.

## Process description

**Fig. 1:**
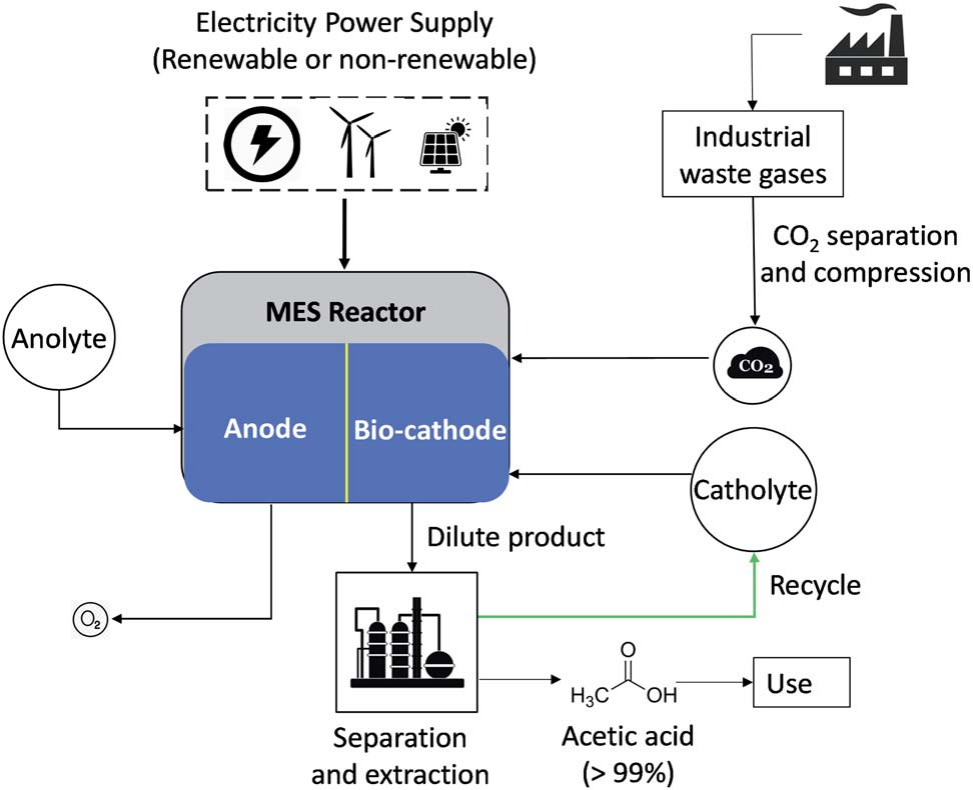
Schematic representation of AA production in MES reactor system where energy is supplied from either renewable or non-renewable sources and CO_2_ is separated from industrial waste gases.

Fig. 1 illustrates the flowchart of the proposed MES plant for producing AA. The plant will employ a series of MES stack where CO_2_ would be bioelectrochemically reduced to AA. For illustration, the process in Fig. 1 is described based on one MES reactor.

### MES reactor

Each MES reactor consists of 2 chambers, one for each electrode, which are immersed in the respective microbial growth medium and separated by an ion-exchange membrane. The production process involves water oxidation at the anode, which generates protons and electrons. The electrons travel in the external circuit, while the protons pass through the ion exchange membrane to the cathode chamber. At the cathode (biocatalyzed), the electrons and protons facilitate the microbial reduction of CO_2_ to organic chemicals such as acetic acid, formic acid, *etc.* This reaction is, however, non-spontaneous and requires an external input of power. In addition to the electric grid, the power required for MES can also be harvested from renewable energy sources (solar, wind). For the analysis, it is assumed that CO_2_ used in the process is captured from industrial waste gases and transported 100 km to the MES plant, and then compressed and stored on-site for continuous use. Herein, a transport distance of 100 km is an approximation, and this would vary depending on the actual plant location.

### Acetic acid extraction

The product obtained from the cathode chamber of MES is in dilute form and needs further separation and extraction. Traditionally, purification of acetic acid has been a challenge, as water and AA have very similar volatilities. Therefore, advanced distillation approaches such as azeotropic distillation, liquid–liquid extraction, extractive distillation, *etc.*, are recommended.^8^

Specific extraction methods such as membrane electrolysis, membrane liquid–liquid extraction or use of ion-exchange resins, that can be retrofitted with MES, have also been studied.^28–31^ And although, technical feasibility of such approaches has been tested at laboratory scale, they need further optimization for improving extraction efficiency on large scale.

**Fig. 2:**
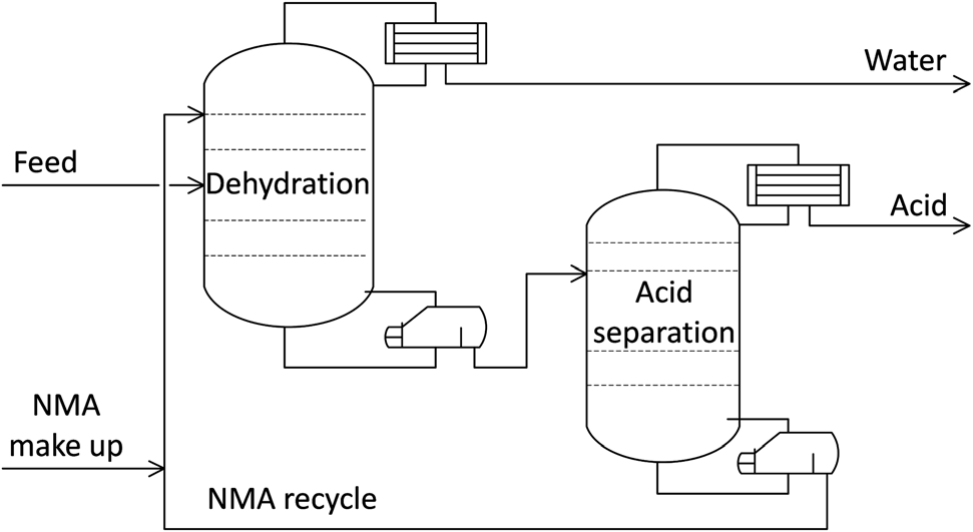
Process flow diagram of the extractive distillation scheme with NMA used as the solvent.

For this study, it was assumed that the dilute product from the MES reactor undergoes extractive distillation using the solvent *N*-methylacetamide (NMA), to separate AA (99.7 wt% purity) and water. Concentrated AA can then be stored for further application, while the water is recycled back into the system. The distillation scheme used in the analysis was based on the work of You *et al.*^32^ and is depicted in Fig. 2. The least volatile component, water, is produced at the condenser and NMA is separated at the bottom of the second distillation column, leaving concentrated AA as the distillate. The distillation simulation was carried out in Aspen Plus using the NRTL-HOC method. This method uses the Non-Random Two-Liquid (NRTL) activity coefficient model for the liquid phase, and the Hayden-O'Connell (HOC) equation of state for the vapor phase.

**Fig. 3:**
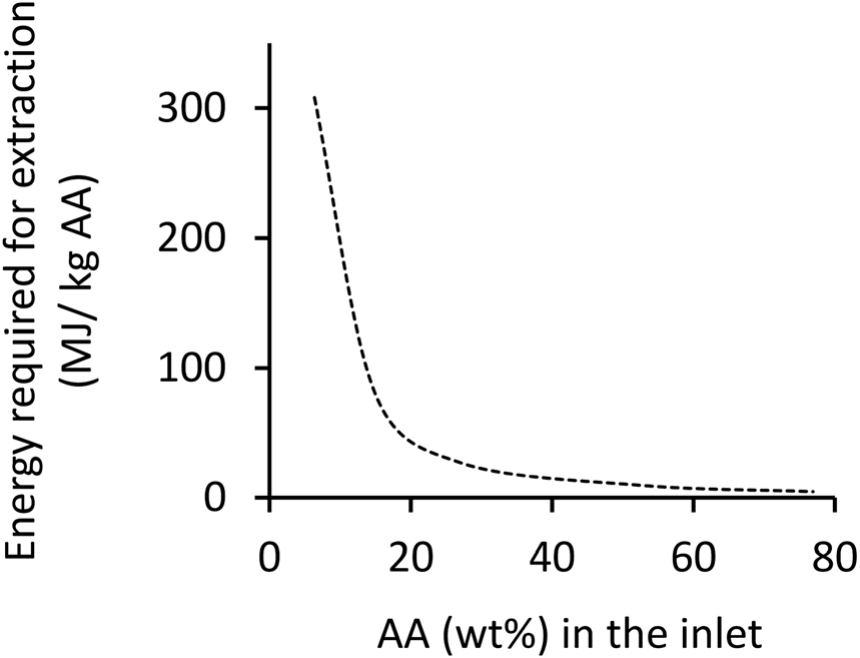
Energy consumption in extractive distillation as a function of inlet feed concentration of AA.

The design duties of the two reboilers were added, from which the reboiler heat associated with 1 kg of AA product was calculated and is presented in Fig. 3. Here it should be noted that the condenser duty was deemed negligible because the coolant fluid (ambient air) was already available, whilst the heating fluid (steam) must be produced (*e.g.* by burning fuel). Also, the pump and fan duties were not included in the calculation of total energy requirement because the pressure was near atmospheric everywhere (except for the steam streams); so, the pumps and fans were to only overcome friction loss and sustain the flow, which was deemed negligible.

Values derived from Fig. 3 are used in impact assessment calculations when deriving the energy consumption for extraction operation which is a function of the outlet concentration of AA from the MES reactor.

## LCA methodology

The LCA is conducted in four phases: (i) goal and scope definition (ii) inventory analysis; (iii) impact assessment and (iv) interpretation, in line with ISO standards: 14040–14044.^33–35^

### Goal and scope of study

The goal of this study is to assess the potential environmental costs and benefits of AA production *via* MES. The scope of the LCA spans from the ‘cradle’ (raw material extraction) to the production gate, before its distribution. For the analysis, we have considered a large-scale stack of MES system based on the reactor described by Aryal *et al.*,^36^ where 3D-graphene functionalized carbon felt composite was used as cathode and had shown enhanced AA productivity on a laboratory scale. The functional unit used to report the environment profile is 1000 t per year of AA produced. Aryal *et al.*^36^ have reported that graphene functionalization of the carbon felt cathode resulted in a two-fold increase in the specific surface area of the cathode, which was effective in increasing the AA production rate by 6.8 times compared to that obtained using unmodified carbon felt cathodes.

Aryal *et al.*^36^'s study represents a very important area of work focussed on modifying or functionalizing electrodes to help enhance MES productivity. So far, such strategies have worked, as demonstrated in the studies by Jourdin *et al.*,^37^ Chen *et al.*,^38^ Cui *et al.*,^39^ Alqahtani *et al.*,^40^ Sharma *et al.*,^41^ Wang *et al.*,^42^ Das *et al.*^43^ and several others. However, functionalization of electrodes requires additional materials and processing (energy), which can have a significant contribution, particularly when considering industrial scale operation. First part of this LCA study assesses whether the benefit of higher production rate using the 3D functionalized cathode outweighs the energy cost and emissions that occur due to the additional processing on an industrial scale reactor. For comparison, MES reactor with carbon felt electrode without any functionalization is also considered.

In addition to this, four different scenarios are considered to determine the required MES performance metrics for sustainable AA production when compared to traditional fossil-based production process. There are several different petrochemical routes for producing AA, but the key processes are, carbonylation of methanol, oxidation of hydrocarbons and oxidation of acetaldehyde. Originally developed by Monsanto, methanol carbonylation has been further improved by different corporations. The process developed by Celanese using rhodium as catalyst contributes the biggest share of worldwide AA production (25%).^44^ For this analysis, environmental impact of MES-based AA production is compared with the Celanese process.

### Life cycle inventory

The inventory used for AA production using MES is based on the primary data (material use, utilities, product yields, *etc.*) obtained in Aryal *et al.*^36^'s study, which is assumed to be scalable to larger operations, generating 1000 t AA per year.

All individual MES systems included a graphite stick anode and Nafion ion-exchange membrane. The current collector (CC) was assumed to be made of copper. Inventory data for carbon felt (CF) cathode was adduced from the work of Minke *et al.*^45^ For the 3D graphene functionalized carbon felt (3D-GFCF), no pre-existing inventory database is available, therefore, a new dataset was generated, following the production process details described by Aryal *et al.*^36^

**Fig. 4:**
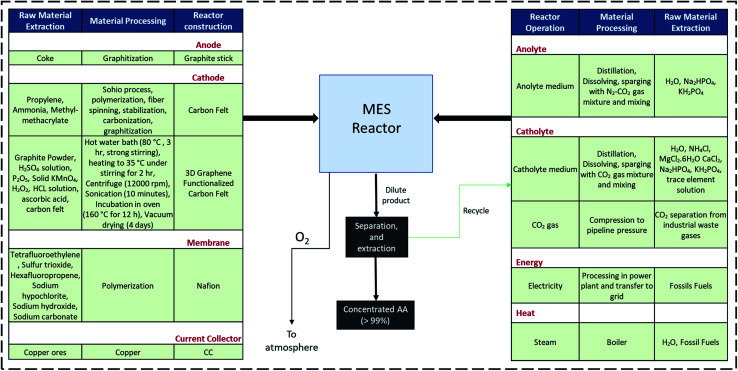
Raw material extraction, MES components manufacturing and reactor operations within the LCA system boundary.

The system boundary, encompassing raw material extraction, material processing of MES components and operating inventory, is illustrated in Fig. 4. Here the details for both types of cathode are provided; either one of them is used depending on the type of MES system being considered. Detailed information on the data sources used for the different inputs and utilities included in this study are summarized in the ESI (Tables S1 and S2†).

Reactor components suffer deterioration and biofouling after long-term operation in contact with microorganisms. Presently, there are not many systematic long-term studies on the material degradation in MES, and hence it is difficult to predict how the electrodes/membrane/CC would behave in large-scale units under extended duration.^17,18^ It is generally possible to clean and regenerate electrodes and membranes in standard electrochemical cells, however, in case of MES, after continuous exposure to bacteria, regeneration would be more strenuous.^46,47^ Also, the chemicals and cost associated with regeneration and maintenance would need to be accounted. Long-term (10 months) operation has been reported with mix culture platform, however for this analysis, life span of reactor components used in each batch was conservatively assumed to be six months.^48^

Catholyte medium consisted of small quantities of salts (ammonium, magnesium and calcium) and phosphates (sodium, potassium) in distilled water, whereas anolyte medium only included phosphates.^37^ Ecoinvent LCI database version 3.6 (ref. 49) was used to extract all the background life cycle inventory data, including the data for the fossil-based AA production (Celanese process). As the modeled MES plant location is assumed to be UK, data specific for electricity production mix, chemicals, and other secondary data was based on UK or European Union averages, when available.

Certain assumptions have been made in the LCI to simplify the analysis. Plant infrastructure, including the reactor framework and other large equipment, are considered to have an extended life and therefore environmental impacts with regards to manufacturing of the infrastructure have not been included.^50^ Also impacts of the transportation of raw materials and supplementary chemicals to the pilot plant, and the production of the biocatalyst, are assumed to be negligible.^26,51^ The dilute product which leaves the MES reactor has certain percentage of solid biomass and dissolved salts from the medium. Before this mixture can be sent to the rectification unit, it needs filtration. The energy consumed in this intermediate filtration process is negligible compared to other processes and hence not included in the analysis.^26^

### Life cycle impact assessment (LCIA)

The life cycle impact assessment (LCIA) methodologies adopted for this study were single-issue LCIA methods, namely, Cumulative Energy Demand (CED) (v 1.11) and IPCC 2013 GWP 100a (v 1.03), as implemented in SimaPro 9.1. CED describes “total quantity of primary energy which is necessary to produce, use and dispose a product”, and provides characterization factors for the energy resources (non-renewable and renewable). IPCC 2013 GWP 100a was developed by the Intergovernmental Panel on Climate Change (IPCC) and describes the global warming potential with a time horizon of 100 years. Results are presented based on two impact categories calculated from the above methods, GHG emissions and non-renewable energy use (NREU), expressed as kg CO_2_ equivalent and MJ of primary units of fossil energy resource depletion per functional unit, respectively.

## Results and discussion

### Contribution analysis

For the first part of the analysis, the environmental impact of producing AA *via* MES using two different types of cathodes, CF and 3D-GFCF, was compared. The production rate, applied voltage and the coulombic efficiency of the two reactors were based on the results reported by Aryal *et al.*^36^Table 1 shows the respective input values per kg AA production and the corresponding energy requirements for the two MES reactors.

**Table 1:** Material and energy requirements per kg AA for producing 1000 t per year AA in the MES plant

Type of MES reactor	Production rate, g m^−2^ d^−1^	Cathode, g	Anode, g	Nafion, m^2^	Current collector, g	Catholyte/anolyte, m^3^	Energy (operation), kW h	CO_2_ capture, kW h	AA wt% from MES, %	Extraction, MJ
CF	2.04	4.46	2.69	1.69	1.28	1.82	3.04	0.39	0.06	>300
3D-GFCF	13.68	0.3	0.4	0.25	0.19	0.27	2.8	0.39	0.37	>300

As can be seen in Table 1, even though use of 3D-GFCF cathode helps in enhancing the production rate, the final AA concentration (wt%) of the output from the MES reactor is still very low (less than 1%). With such low inlet feed, extraction is impractical and unsustainable due to the astronomical energy requirements (>300 MJ kg^−1^ AA), as can be seen from Fig. 3. When the production rate is increased (as discussed in the next section), the final AA concentration (wt%) that serves as inlet to the extraction unit, also increases. This helps in reducing the energy requirement for separation and purification. For this part of the analysis, focus was on understanding the contributions of other subprocesses towards GHG emissions and NREU, excluding extraction.

**Fig. 5:**
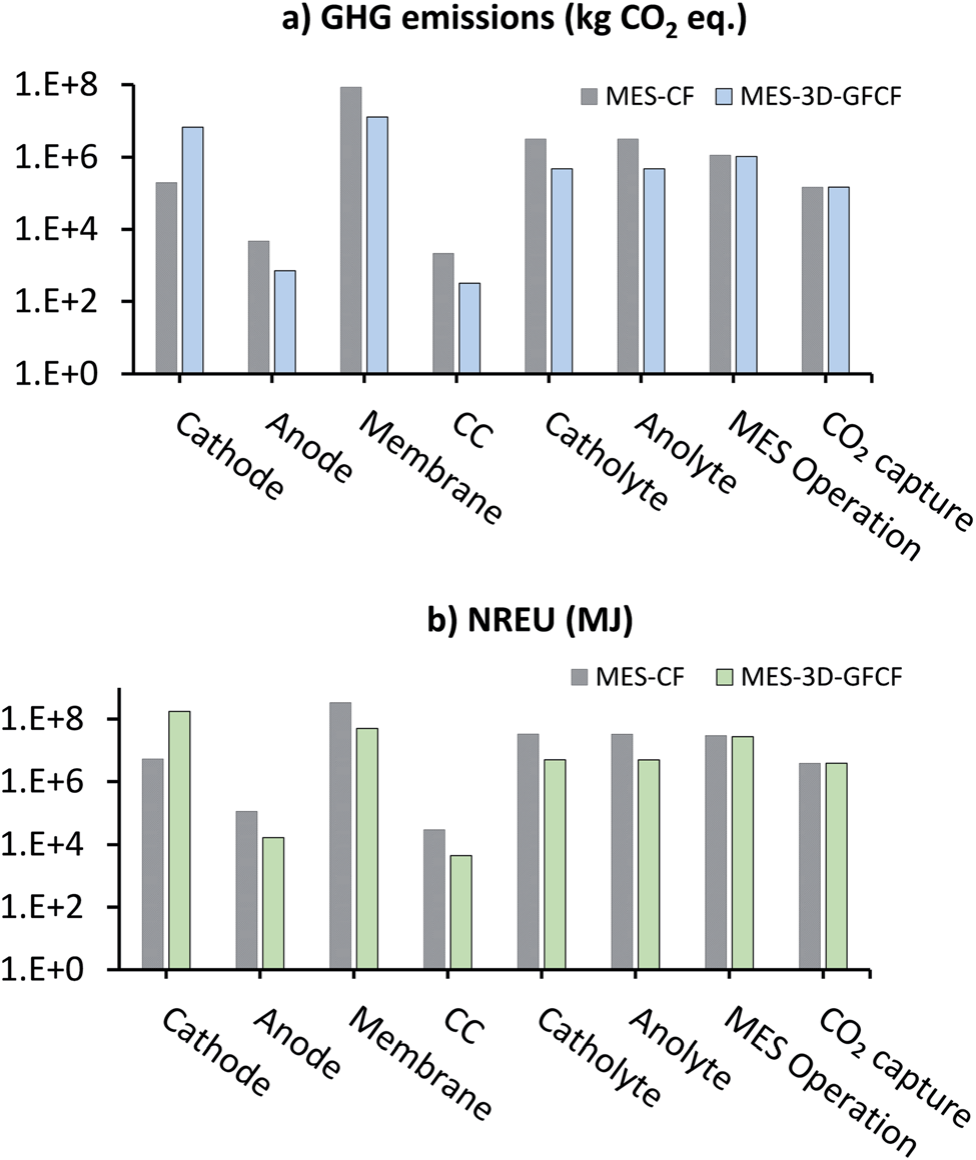
Breakdown of subprocess contributions of (a) GHG emissions (kg CO_2_ eq.) and (b) NREU (MJ) for 1000 t per year AA production from MES using either CF or 3D-GFCF as cathode.

Fig. 5 shows the comparison of individual subprocess contributions of GHG emissions (kg CO_2_ eq.) and NREU (MJ) for producing 1000 t per year AA in the MES plant using either CF or 3D-GFCF as cathode (data for this chart is provided in ESI Tables S3 and S4†). Looking at individual contributions it can be seen that production of 3D-GFCF cathode leads to more than 40 times GHG emissions and NREU when compared to the production of standard CF cathode. This is expected as preparation of 3D-GFCF requires additional processing and chemicals, which lead to the higher energy requirements and subsequently more emissions. However, except for cathode, for all other components and utilities, MES with CF cathode leads to more GHG emissions and NREU in comparison to MES with 3D-GFCF cathode.

Fig. 5 also shows that for both MES-CF and MES-3D-GFCF systems, membrane is one of the biggest contributors to emissions and energy use (except for cathode NREU for MES-3D-GFCF). This is primarily because we have assumed, Nafion as the ion-exchange membrane in this analysis, and manufacture of which is an energetically expensive process. Membrane is thus highlighted as a key environmental hotspot, and efforts should be focussed on optimizing and improving the performance of suitable alternatives such as SPEEK, Ultrex, Selemion, *etc.*,^52–55^ so that these other options can achieve equivalent or enhanced performance compared to Nafion. Other than membrane and cathode; medium used in the reactor (catholyte and anolyte) also has major contribution to GHG emissions and NREU, particularly for MES with CF as cathode. This is because of the large quantities of medium required for MES using CF, due to its low productivity. For MES-3D-GFCF systems, energy used during operation is a bigger contributor to the impact categories than the medium. This is followed by energy consumed in CO_2_ capture, and anode production, and then finally CC production which has the lowest impact in both the categories.

Net GHG emissions and NREU for the two systems can be calculated by combining the individual contributions of the different subprocesses. Net calculation of GHG also includes the emissions savings due to fossil-CO_2_ consumption in AA (not shown on the log–log plot in Fig. 5a, as the values are negative). For MES with CF cathode, the net GHG emissions (9.14 × 10^7^ kg CO_2_ eq.) were 4.5 times more than that when using GFCF cathode (2.02 × 10^7^ kg CO_2_ eq.). Total NREU for MES with CF cathode (4.43 × 10^8^ MJ) were also higher (about 1.6 times) than that compared with MES using GFCF cathode (2.67 × 10^8^ MJ). These results show that even though modification or functionalization of cathodes is energy intensive, when it helps to improve the productivity substantially (as in the current case), the energy and emission savings on all other inputs compensate for the supplemental energy in production of functionalized cathodes. Depending on the total productivity gain, modified cathodes have the potential to reduce the overall environmental impact of MES systems.

Future research on MES also needs to focus on effective regeneration and reuse of reactor components after biofouling and deterioration. If the total lifespan of components can be extended to few years, this can significantly reduce the amount of material used and thus mitigate the associated environmental impact.

### Scenario analysis

As seen from the above analysis, production rate of AA from MES reactor has to be significantly high before it becomes pragmatic to extract AA (using conventional rectification methods) for further use. In this section, we discuss alternative scenarios and calculate their environmental impacts to identify, when, if it is possible in future, MES systems can become environmentally cleaner or even equivalent to the current fossil-based routes of AA production.

#### Scenario 1

One of the highest area-specific production rates of AA from MES systems^56^ reported so far is 685 g m^−2^ d^−1^. However this rate is obtained with MES operating in fed-batch mode. For commercial scale production of bulk chemicals, continuous mode of operation is preferred. At present, the success rate of continuous MES systems is as expected, much lower than batch and fed-batch systems.^15^

Even with 685 g m^−2^ d^−1^ production rate, the reported product titer is only 11 g L^−1^ which is too small to justify extraction, particularly considering the vast energy requirement. Therefore, for the range of production rates and final concentrations of AA reported in literature, the extraction part alone would completely overlap all other factors in any sustainability assessment study. Such an analysis can only be performed for scenarios assuming more productive MES system than what have been reported so far.^24,26^

For a future MES reactor with enhanced acetogenesis, inspiration can be drawn from the results reported by Kantzow *et al.*,^57^ who have demonstrated AA production at space time yield of 148 g L^−1^ d^−1^ from H_2_:CO_2_ mixtures in a continuous gas fermentation reactor using *Acetobacterium woodii*. These results are promising and show that under optimized reactor conditions, it is theoretically possible to improve production of AA using acetogenic bacteria significantly.

To assume a high productivity MES system as the first scenario in this LCA analysis, reactor from the current system with 3D-GFCF as cathode was assumed to operate in a continuous mode and reach a space time yield of 148 g L^−1^ d^−1^. To account for such a significant improvement, the current system operating in continuous mode was required to deliver much higher area specific production rate. Accordingly, the electrode and membrane area and media volume requirements would also change, which are tabulated in the ESI (Table S1†). The output from the continuous MES reactor was forwarded to the rectification unit for AA extraction. Energy consumption for extraction was calculated based on process simulation results as described earlier. It should be noted that compared to a fed-batch system (which is discussed as a separate scenario later), the production rate requirement was typically much higher for a continuous system to achieve the same product concentration.

**Fig. 6:**
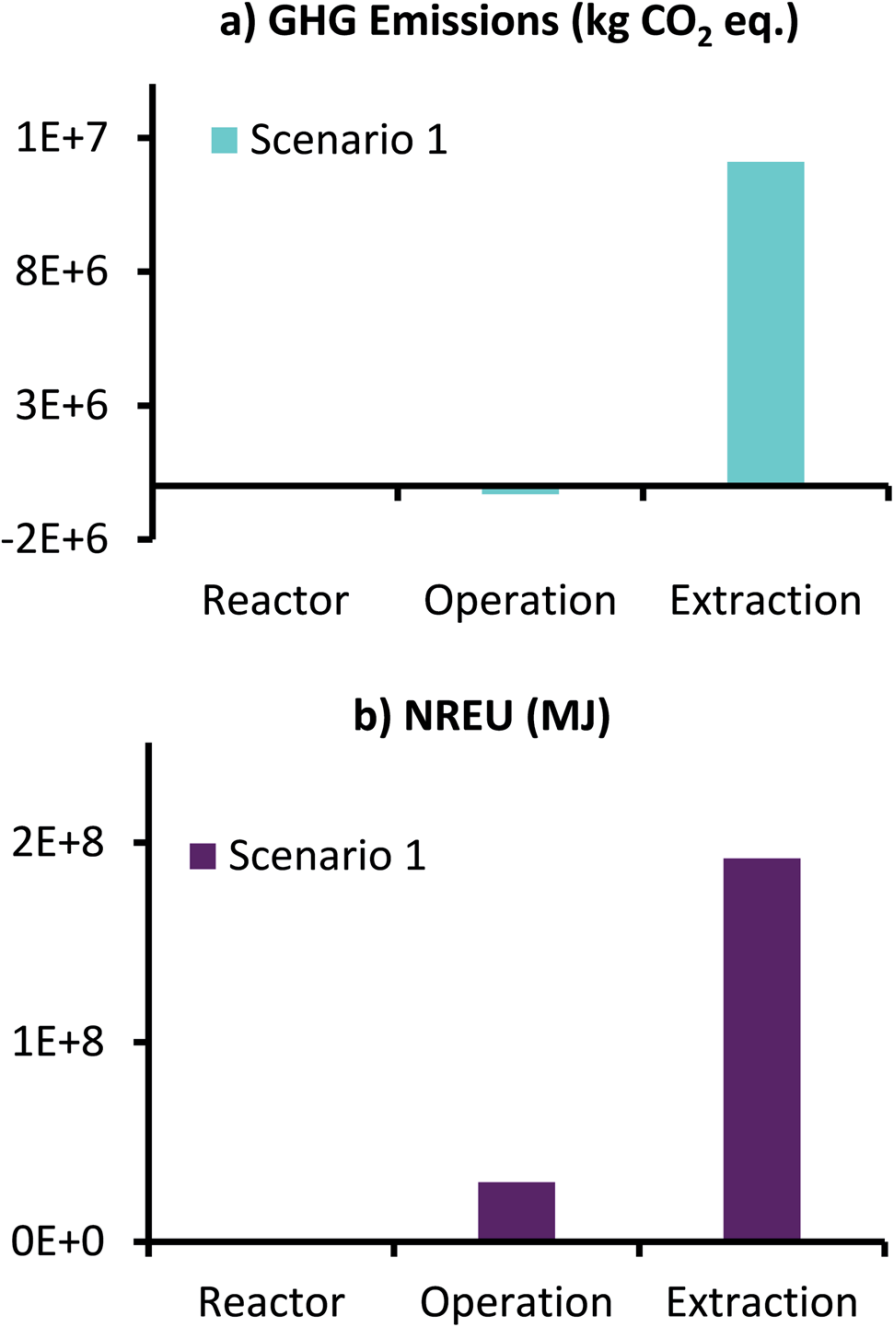
(a) GHG emissions (kg CO_2_ eq.) and (b) NREU (MJ) for production of 1000 t per year AA using MES with 3D-GFCF as cathode and space time yield of 148 g L^−1^ d^−1^.

Environmental performance results of this scenario in terms of GHG emissions and NREU for producing 1000 t per year AA from MES, are presented in Fig. 6. Here the two impact categories for the different inputs for reactor components and operation are collated and presented together. Therefore, the label, ‘Reactor’ represents the sum of corresponding values for anode, cathode, CC and membrane, whereas the label, ‘Operation’ refers to sum of corresponding values for anolyte, catholyte, CO_2_ utilization, and energy for MES operation and CO_2_ capture. ‘Extraction’ is represented individually (data for all components is provided in ESI Tables S5 and S6†).

As can be seen from Fig. 6, for scenario 1, even with the high production rate, extraction still dominates both GHG emissions and NREU. These results are in accordance with those reported by Christodoulou *et al.*^26^ for AA production using MES. It should, however, be noted that the increased productivity results in lower use of catholyte medium, which combined with the environmental credit for CO_2_ utilization, helps in achieving net negative GHG emissions for MES operation (−3.12 × 10^5^ kg CO_2_ eq., Fig. 6a). This is encouraging as it suggests that if an alternative, less energy intensive separation technology can be developed for AA extraction, the total GHG emissions can be comparable to the traditional petrochemical route of AA production. In terms of non-renewable energy demand, reactor manufacturing requires the least energy, followed by ‘Operation’ and ‘Extraction’. NREU for operation is more than 300 times of that required for ‘Reactor’, whereas, ‘Extraction’, alone requires close to 6.5 times more energy that the combined energy required in the reactor manufacture and continuous operation over an year. This highlights the significant influence of extraction on the overall production process.

To understand MES sustainability in a broader sense, environmental impact for MES-based AA production from scenario 1 was compared to the AA production using the fossil-based Celanese process. As the publicly accessible data on the industrial Celanese process is limited, environmental impact for the fossil-derived AA production was calculated based on the corresponding data available in the Ecoinvent database.^49^

**Fig. 7:**
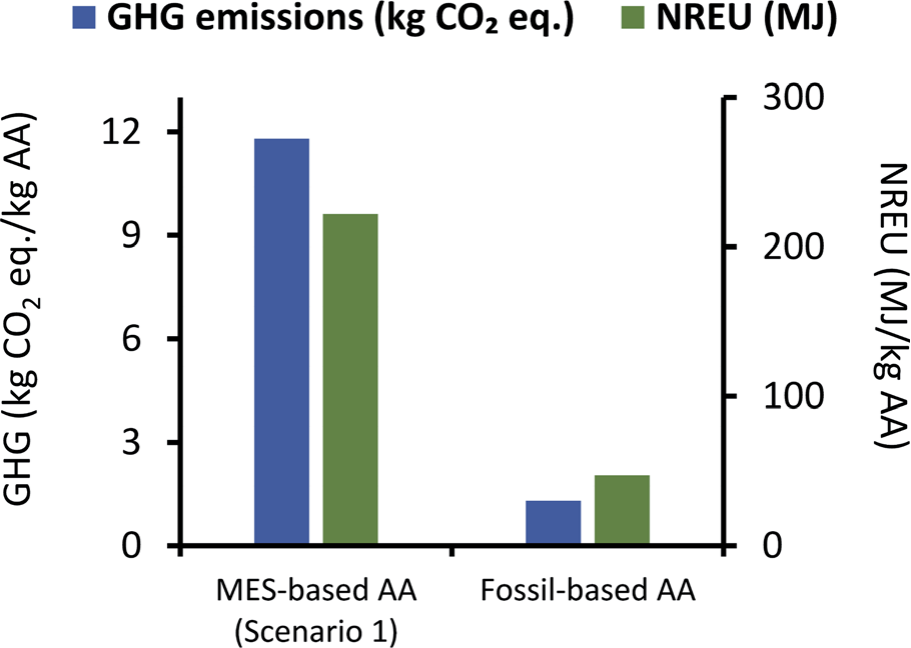
Comparison of GHG emissions (kg CO_2_ eq.) and NREU (MJ) per kg AA production using MES scenario 1 and the fossil-based process.

It can be seen from Fig. 7 that net GHG emissions and NREU per kg AA using MES as per scenario 1, are 11.8 kg CO_2_ eq. and 221.89 MJ, respectively. These values are comparable to impact parameters obtained by Christodoulou *et al.*^26^ (when considering same elements in the system boundary), who have reported net energy consumption of 149.35 MJ and GHG emissions of 5.82 kg CO_2_ eq. for each of kg AA produced *via* MES. The slightly lower values can be explained by the fact that Christodoulou *et al.*^26^ had assumed a higher product titer, which reduces the energy requirement for extraction. It should also be noted that Christodoulou *et al.*^26^ have provided the net energy consumption which includes both renewable and non-renewable energy, so the NREU component may be lower. Okoroafor *et al.*,^27^ who had also assumed much higher product titer than that used in the current work, reported the net energy consumption for AA production *via* MES to be 95.84 MJ kg^−1^ AA. This value is less than half of that obtained in the current study, however, it is not clear from Okoroafor *et al.*^27^'s results why energy consumption reduces further, even after adding the energy load at anode.

Fig. 7 also shows that GHG emissions and NREU per kg of AA for MES-based system are about 10 times and 4.7 times higher, respectively, when compared to AA production using the fossil-based process. These results clearly indicate that even if the MES productivity is assumed to be the highest value reported for acetogenic bacteria,^57^ the system would still be far from providing a sustainable alternative to the current fossil-based AA production process. This is largely attributed to the excessively high energy demand during extraction.

If a blended approach is used for separation, where AA is first extracted *in situ* using an additional chamber or ion-exchange resins, and then forwarded to rectification units for extractive distillation or hybrid extraction/distillation, it may be possible to lower the energy requirement and thus improve the environmental performance of MES. Based on the analysis for scenario 1, if the energy used for extraction can be lowered to 15.3 MJ kg^−1^ AA, the total environmental impact of MES systems can become comparable to that from current fossil-based processes. An alternate solution to circumvent this issue is discussed next.

#### Scenario 2

MES was originally aspired as a technology that can help to store the excess renewable energy (solar, wind, *etc.*) in the chemical bonds of multi-carbon compounds.^3,58^ Considering that the net zero carbon target is imminent upon us, technology like MES that can help synthesize chemical products by CO_2_ reuse could be a way to store both renewable energy as well as carbon. Therefore, for the second scenario, the influence of source of electricity that is used in the process was investigated. For all the previous cases, the UK power grid distribution was used for electricity inputs to the unit processes. The share of electricity technologies on this production mix, are valid for the year 2016, as described in the Ecoinvent v 3.6. database.^49^ In the second scenario, source of electricity for the MES plant was changed from UK production mix to grid-connected low voltage electricity from open ground photovoltaic (PV) plant in the UK. The source of the data was again, Ecoinvent v 3.6. database. Except for electricity origin, all other values, including the production rate of AA, were assumed to be similar to that of scenario 1.

**Fig. 8:**
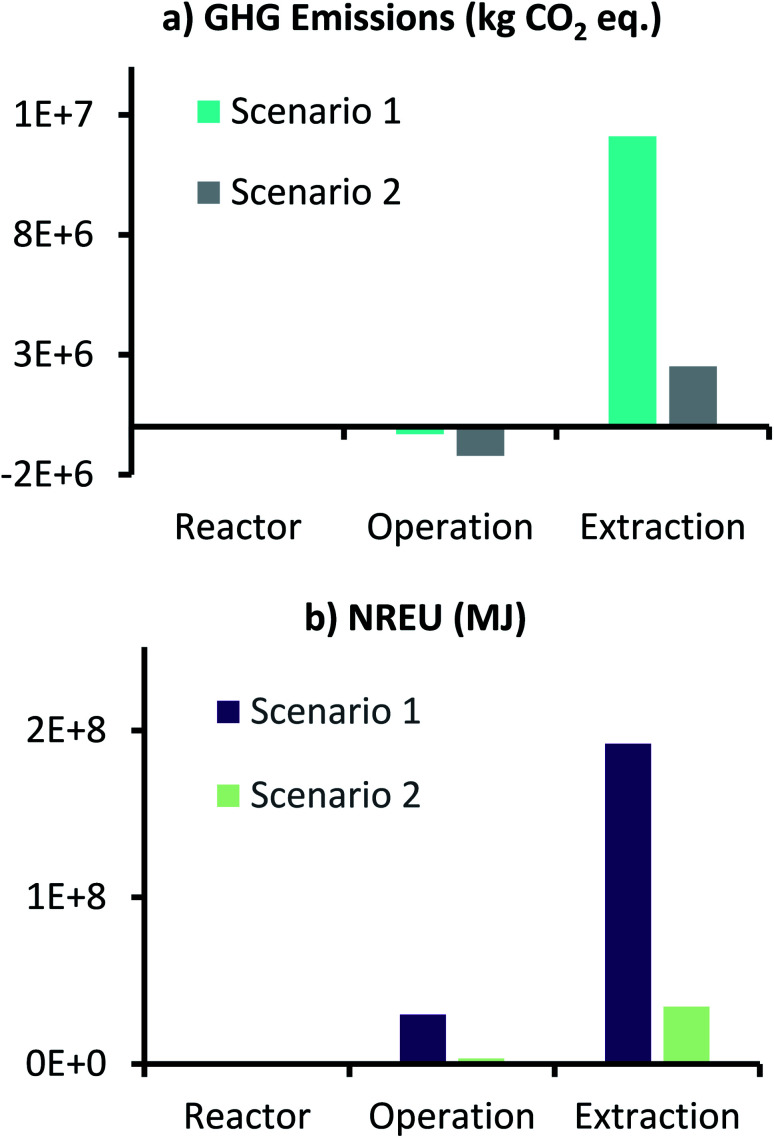
GHG emissions (kg CO_2_ eq.) and NREU (MJ) for production of 1000 t per year AA using MES with 3D-GFCF as cathode for scenario 2.

Fig. 8 shows the GHG emissions and NREU for production of 1000 t per year AA from the MES plant, where electricity used in the process was generated from renewable (solar) source. For comparison, scenario 1 results are also included in Fig. 8. As anticipated, net GHG emissions (1.31 × 10^6^ kg CO_2_ eq.) and NREU (3.8 × 10^7^ MJ), both dropped substantially (about one order of magnitude) when compared to scenario 1 (1.18 × 10^7^ kg CO_2_ eq. and 2.2 × 10^8^ MJ). This decrease in emissions and energy use was more pronounced for ‘Operation’ and ‘Extraction’ sub-categories, which involve higher energy usage as opposed to the reactor components. Compared to scenario 1, GHG emissions for scenario 2 ‘Operation’ were 4 times lower (thus more savings), and that for ‘Extraction’ were about 4.8 times lower. Similarly, NREU for ‘Operation’- scenario 2 was 8.8 times lower and ‘Extraction’-scenario 2 was 5.6 times lower, as compared to scenario 1.

**Fig. 9:**
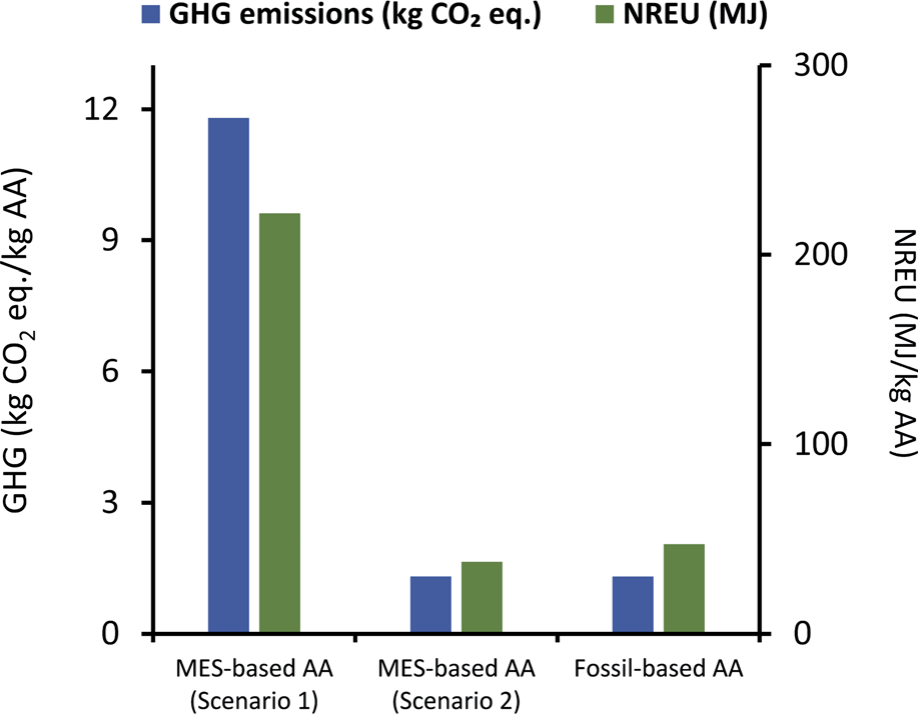
Comparison of GHG emissions (kg CO_2_ eq.) and NREU (MJ) per kg AA production using MES scenario 1 & 2, and the fossil-based process.

However, in order to understand if use of renewable solar energy can make MES-based AA production more attractive compared to traditional methods, environmental impact of scenario 2 was also compared to fossil-based AA production (Celanese process), and the results are described in Fig. 9. Here results of scenario 1 are also included for reference. As can be seen from Fig. 9, use of renewable (solar) energy, brings both emissions and the energy demand of MES plant much closer to that of fossil-based process. In terms of absolute values, GHG emissions and NREU per kg AA for MES plant as per scenario 2, are 1.31 kg CO_2_ eq. and 37.9 MJ, which now became comparable (and even cleaner when comparing NREU) to the same from fossil-based process (1.3 kg CO_2_ eq. and 47.3 MJ per kg AA). This result is significant as it shows that MES systems have the potential to become equivalent to the petrochemical route for AA production. The one major issue however, would be the supply of such high demand of renewable (solar) energy throughout the year (this includes 32.5 kW h kg^−1^ AA for extraction alone in the present scenario). This would possibly involve setting up a photovoltaic (PV) electricity production facility near the MES plant and large batteries to store power, which is a huge investment and would severely restrict the large-scale implementation of MES for continuous AA production. This major bottleneck can only be solved by either increasing the production rate and the product titer further or to find an extremely-efficient and less energy intensive method for AA extraction.

Continuous operation as shown in the two scenarios is big hindrance for MES systems, as this mode makes it more difficult to boost product concentration. Next two scenarios discuss an alternate approach.

#### Scenario 3 and 4

While continuous mode of operation is common in fossil-based chemical industries, for bioprocess industries, fed-batch mode, which offers more control over the microorganisms' growth, is sometimes preferred. Fed-batch operation supports microbial accumulation and allows high biomass density, and therefore bioprocesses where product yield is a function of microbial growth, prefer fed-batch processing. As all these conditions are also true for MES, many of the studies reported in literature have demonstrated AA production in a fed-batch operation. One major drawback of using fed-batch systems for large scale production of chemicals is the excessive material consumption for reactor manufacture and operation.

MES systems with fed-batch mode have been reported with individual cycles as small as few days and some studies with single run lasting even up to 10 months.^48,56,59^ Continuing the operation longer has the obvious advantage of higher final product concentration, but this can lead to several complications as well, which prevents longer cycle times. One major issue is the increased build up of inhibitors or toxins, which can lead to reduced productivity. Longer run and changing operating conditions with product build-up, sometimes also leads to side reactions, or chain elongation, which may reduce the final concentration of the required product.

Considering that the rate of production in MES is directly correlated to microbial concentration, and the difficulties in keeping constant cell population density in continuous systems, fed-batch systems could be seen as an alternative even for large scale operation. Thus, it is worth considering what will be the environmental impact if the MES stack is run in fed-batch mode. This possibility was evaluated in scenario 3. Here, in order to maintain a continuous production, it was required to operate two set of systems in parallel, so that once the cycle for one system was complete, the other could continue production without gap. To keep it consistent with previous two scenarios, the system was assumed to have the same final productivity as the continuous mode but here the cycle time was assumed as 30 days. It is presumed that there is consistent microbial growth with no build-up of toxins or inhibitors up to 30 days. This cycle time will vary based on the microbial culture and operating conditions. It should be noted that much longer operation cycle times have been reported for fed-batch MES systems.^15^

Complimentary to scenario 2, effect of renewable energy source was also evaluated for the fed-batch operation, as scenario 4, where all operating conditions were similar to scenario 3 (fed-batch mode), except the power, which was supplied from a PV facility instead of grid.

**Fig. 10:**
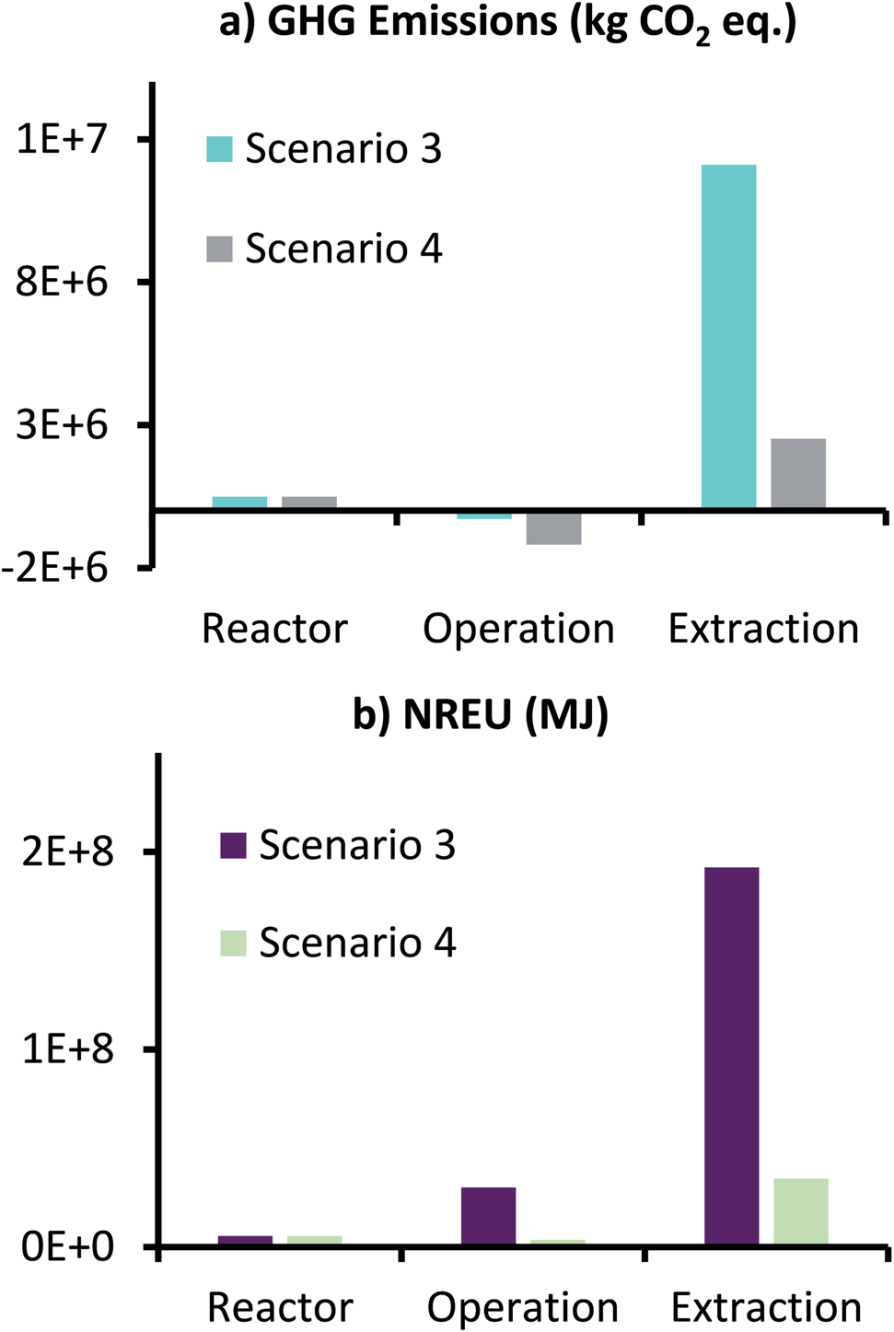
GHG emissions (kg CO_2_ eq.) and NREU (MJ) for production of 1000 t per year AA using MES with conditions described for scenario 3 and 4.

Impact assessment results of scenario 3 and 4 are presented in Fig. 10, which show GHG emissions and NREU for production of 1000 t per year AA. Net GHG emissions in scenario 3 and 4 were 1.23 × 10^7^ and 1.8 × 10^6^ kg CO_2_ eq. respectively, while NREU in these two scenarios was 2.28 × 10^8^ and 4.37 × 10^7^ MJ. This shows that changing the mode of operation from continuous to fed-batch results in slight increase in GHG emissions, about 4.3%, when comparing scenario 1 to 3, and same when comparing scenario 2 with 4.

Looking at individual contributions of GHG emissions, it can be seen that the ‘Reactor’ contributions which were negligible in continuous operations, have now increased and account for around 4% of the total emissions for scenario 3 and 27% of the total emissions in scenario 4. This is primarily due to the additional material and energy requirements due to the increased number of reactors that are required for fed-batch operation. NREU for ‘Reactor’ also increased, and now accounts for about 2.5 and 13% from the total NREU of scenario 3 and 4 respectively. Considering the productivity was assumed to be same as that of scenarios 1 and 2, there is not major difference in impact for ‘Extraction’ because of fed-batch operation in scenario 3 and 4.

**Fig. 11:**
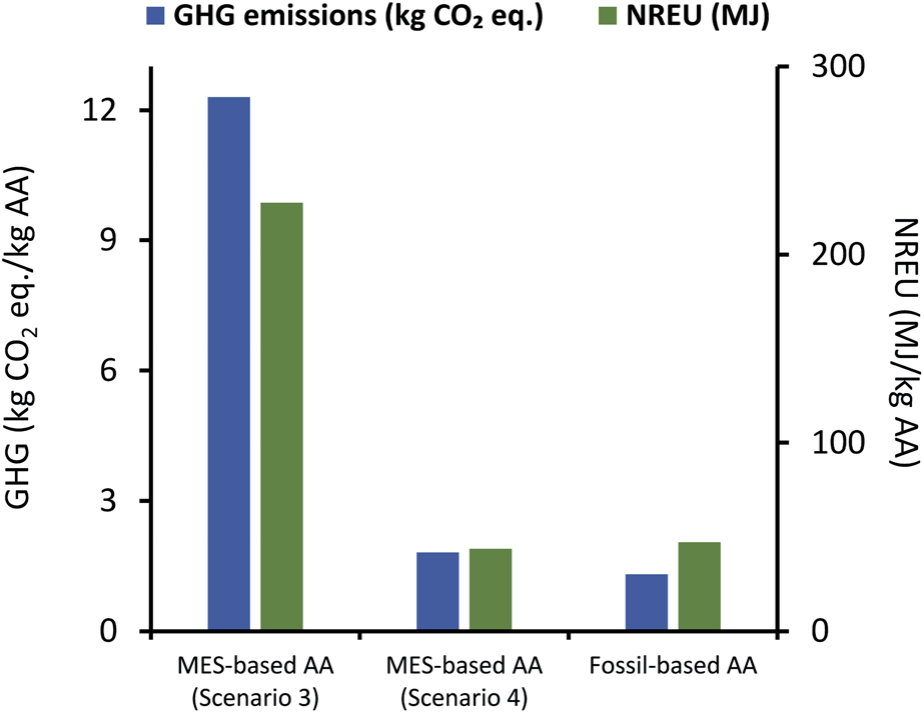
Comparison of GHG emissions (kg CO_2_ eq.) and NREU (MJ) for 1 kg AA production using MES scenario 3 & 4 and the fossil-based process.

Emissions and non-renewable energy requirements per kg AA for MES-based production as per scenario 3 and 4 were compared with fossil-based production process, and these results are presented in Fig. 11. As can be seen here, results for scenario 3 and 4 are similar to the comparison between scenario 1 and 2 with fossil-based process (Fig. 9), except for the fact that there is slight increase in both GHG emissions and NREU.

For scenario 4, NREU per kg AA production (43.7 MJ) is higher when compared to the same for scenario 2 (37.9 MJ) but is still 7.5% smaller than that for fossil-based process (47.3 MJ), while GHG emissions (1.8 kg CO_2_ eq. per kg AA) are about 40% higher than the corresponding value for the fossil-based process. Similar to continuous mode, the fed-batch system is unable to deliver better or even comparable environmental performance as fossil-based method, even after assuming a significant increase in MES productivity. Performance only becomes comparable when all energy used in the process is derived from a renewable source (as shown for results of scenario 4), which would remain a major roadblock for large scale implementation and expansion to multiple locations. Results from the four scenarios are compiled and presented in Table 2 for easy comparison.

**Table 2:** Comparison of the four scenarios and the fossil-based process for AA production

	Process	Space time yield, g L^−1^ d^−1^	Electricity source	GHG emissions, kg CO_2_ eq. per kg AA	NREU, MJ kg^−1^ AA
Scenario 1 (continuous mode)	MES with 3D-GFCF	148	GB production mix	11.80	221.89
Scenario 2 (continuous mode)	MES with 3D-GFCF	148	Photovoltaic plant	1.31	37.95
Scenario 3 (fed-batch mode)	MES with 3D-GFCF	148	GB production mix	12.30	227.68
Scenario 4 (fed-batch mode)	MES with 3D-GFCF	148	Photovoltaic plant	1.81	43.74
Fossil-based system	Celanese process			**1.31**	**47.26**

Kantzow *et al.*^57^'s promising results based on *Acetobacterium woodii* show that it is possible to increase productivity by optimizing reactor configuration and reaction conditions. Therefore, as a theoretical analysis, a threshold value of minimum production rate can be estimated, which will have an environmental impact lower than the fossil-based AA production process, without the use of renewable energy. For the MES system operating in fed-batch mode, this threshold value is reached when the production rate exceeds 4100 g m^−2^ d^−1^. The scale of this value highlights the improbability of achieving purified AA sustainably using a fed-batch MES when combined with the extraction approach used in this analysis. Considering the best production rates obtained from MES systems currently (685 g m^−2^ d^−1^), the proposed threshold value appears to be quite high and an ambitious target. Also, such a threshold production rate would be much higher for a continuous MES. However, it should be noted that these values are only based on the current system with the respective assumptions. Threshold values would depend on the particular MES configuration, electrodes, microbiome used at the cathode, and the type of approach used for extraction. Also, if part of the energy required for the operation and extraction can be supplied from a renewable source, the minimum threshold for sustainable performance can be further reduced.

At the current productivity MES based AA production is not sustainable from an environmental perspective as shown in this analysis or even from an economic point of view, as has been shown previously.^60–62^ Reaching the proposed threshold production rate using available electrode materials, biocatalysts, and reactor configurations, while maintaining the required system efficiency, is extremely challenging and at present almost inconceivable in either continuous or fed-batch mode. However, these values are not presented to dissuade researchers from investigating AA production *via* MES, but to refocus research efforts.

Considering the bottlenecks in increasing the productivity of MES and the high energy requirement for extraction, sustainable use of AA produced *via* MES could ideally be focussed in applications where the dilute AA from the reactor can be employed directly. This could involve applications where genetically engineered microorganisms are used, that can accept AA in low concentrations and convert them to medium-chain carboxylic acids or other valuable compounds.^60,63,64^ Such chain elongation reactions may involve use of new synthetic biology approaches and could be carried out in detached fermentation reactors outside the MES system.

Simultaneously, efforts could be continued for finding suitable options for less energy intensive extraction of AA. These approaches, some of which are already being investigated include, effective use of membrane technology and employing an additional middle chamber, use of ion-exchange resins, *etc.*, or combination of technologies to modify MES reactor configuration in ways that can actively segregate the dilute AA, allowing *in situ* extraction over time. No matter which approach is pursued, it would be critical to not only monitor the economic feasibility but also the environmental implications of such new strategies.

## Conclusions

This study presented a cradle-to-gate LCA analysis of microbial electrosynthesis assuming a functional unit of 1000 t per year AA production. Results from the contribution analysis showed that other than separation of AA, membrane and reactor medium are the other significant environmental hotspots in terms of GHG emissions and non-renewable energy use. LCA analysis, based on comparison between carbon felt cathode with a 3D graphene functionalized version of the same electrode, found that if the enhancement in productivity is significant after modification, the overall environmental impact of the MES can be reduced. However, this also indicates that modification of the electrodes where the energy and material costs are high and the performance improvement is not proportionate, the process may not be environmentally favourable. Thus, it is recommended that new more efficient cathodes being developed for MES, should also be assessed for their environmental performance, before they can be classified as suitable for pilot-scale studies.

A sensitivity analysis, assuming a more productive MES system with space time yield of 148 g L^−1^ d^−1^ is also presented. This analysis was based on four scenarios, which involved either fed-batch or continuous operation, with electricity source as either grid power or a photovoltaic facility. Impact assessment results show that for the assumed high productivity, the environmental impact of AA production using MES could become comparable to traditional fossil-based method, but only if the energy source is completely renewable, which is a significant challenge. Among the two modes of operation, fed-batch processing leads to higher environmental impact, however, the product accumulation helps in achieving the same performance at low production rate as compared to continuous mode.

A threshold production rate for a fed-batch MES reactor that could possibly lead to sustainable AA production was found to be 4100 g m^−2^ d^−1^. While this value can be reduced by using renewable energy for part of the process, based on the current productivity, replacing conventional AA production practices sustainably, appears to be a bridge too far at this stage in MES research. It may be perhaps prudent to divert focus to applications that can use the dilute AA from MES directly for chain-elongation reactions to produce medium chain carbon compounds such as butyric acid, caproic acid, *etc.*, which have higher economic value and are also easier to extract.

## Conflicts of interest

There are no conflicts to declare.

## Supplementary Material

RA-011-D1RA00920F-s001
