# Supplementary material for: Microbial electrosynthesis: is it sustainable for bioproduction of acetic acid?
Source: RSC Adv. 2021 Mar 8;11(17):9921–32. doi: 10.1039/d1ra00920f (PMC8695651; doi:10.1039/d1ra00920f)
Supplement: RA-011-D1RA00920F-s001 [file RA-011-D1RA00920F-s001.pdf]

Electronic Supplementary Material (ESI) for RSC Advances.
This journal is © The Royal Society of Chemistry 2021

Supplementary Information

Microbial Electrosynthesis: Is it sustainable for bioproduction of acetic acid?

Siddharth Gadkari*[a,b], Behzad Haji Mirza Beigi[b], Nabin Aryal[c], and Jhuma Sadhukhan[a,b]

[a] Department of Chemical and Process Engineering, University of Surrey, Guildford GU2 7XH, United Kingdom
[b] Centre for Environment and Sustainability, University of Surrey, Guildford, Surrey GU2 7XH, United Kingdom
[c] Department of Microsystems, University of South-Eastern Norway, Horten, Norway

Life Cycle Inventory

This section presents the life cycle inventory for all the components analysed in the MES plant, for both contribution and scenario analysis.

Table S1: Life cycle Inventory data per kg AA of the different MES reactors used in the analysis for bioproduction of 1000t/year AA:

	Scenario 1 and 2	Scenario 3 and 4
Cathode	3D-GFCF	3D-GFCF
Mode of operation	Continuous	Fed-batch
Dilution rate (h^{-1}) / operation cycle (d)	0.042 (h^{-1})	30 (d)
Space time yield (g L^{-1} d^{-1})	148	148
Cathode (g/kg AA)	0.00013	0.00756
Anode (g/kg AA)	0.00017	0.01001
Nafion (m^2/kg AA)	0.00042	0.02512
Current collector (g/kg AA)	0.00008	0.00476
Catholyte/anolyte (m^3/kg AA)	0.00068	0.00676
Energy (kWh/kg AA)	2.80	2.80
CO_2 capture (kWh/kg AA)	0.39	0.39
AA wt% from MES	12.88	12.88
Extraction (MJ/kg AA)	117.00	117.00

For the catholyte and anolyte, 90% recycle rate has been assumed.

Table S2: Data sources of the different inputs used in the LCA analysis:

Process Input	Data Source
Grid Electricity	Electricity, medium voltage {GB}
Renewable electricity	Electricity production photovoltaic {GB}, 570kWp open ground installation, multi-Si
CF Cathode	Minke et al. 2017
3D-GFCF Cathode	Aryal et al. 2016, Dey et al. 2015
Graphite Stick	Graphite, battery grade {GLO}
Anolyte, Catholyte	Jourdin et al. 2014
Current Collector	Copper {RER}| production, primary
Nafion	Evangelisti et al. 2017
Carbon dioxide, fossil	Ecoinvent data on emissions to air
Oxygen	Ecoinvent data on emissions to air
Acetic acid	Acetic acid production, in 98% solution state {RER}
Heat	Heat, from steam, in chemical industry {RER}

Life cycle impact assessment results

This section presents the life cycle impact assessment data in terms of individual contributions to GHG emissions and NREU from different sub-processes for the case studies investigated under contribution and scenario analysis.

Table S3: GHG emissions (kg CO_2 eq.) of individual subprocesses from production of 1000t/year AA using MES with CF and 3D-GFCF cathode.

	GHG Emissions (kg CO_2 eq.)	
	MES-CF	MES-3D-GFCF
Cathode	1.99E+05	6.81E+06
Anode	4.74E+03	7.06E+02
Membrane	8.49E+07	1.27E+07
CC	2.14E+03	3.19E+02
Catholyte	3.20E+06	4.78E+05
Anolyte	3.20E+06	4.76E+05
MES Operation	1.14E+06	1.05E+06
CO_2 capture	1.48E+05	1.48E+05
CO_2 utilization	-1.47E+06	-1.47E+06
TOTAL	9.14E+07	2.02E+07

Table S4: NREU (MJ) of individual subprocesses for production of 1000t/year AA using MES with CF and 3D-GFCF cathode.

	NREU (MJ)	
	MES-CF	MES-3D-GFCF
Cathode	5.31E+06	1.75E+08
Anode	1.12E+05	1.68E+04
Membrane	3.38E+08	5.03E+07
CC	2.98E+04	4.45E+03
Catholyte	3.34E+07	4.98E+06
Anolyte	3.32E+07	4.96E+06
MES Operation	2.99E+07	2.75E+07
CO_2 capture	3.88E+06	3.88E+06
TOTAL	4.43E+08	2.67E+08

Table S5: GHG emissions (kg CO_2 eq.) from producing 1000t/year of AA using MES as described in Scenario 1, 2, 3 and 4.

		GHG Emissions (kg CO_2 eq.)			
		**Scenario 1**	**Scenario 2**	**Scenario 3**	**Scenario 4**
**Reactor**	Cathode	2.83E+03	2.83E+03	1.70E+05	1.70E+05
	Anode	2.94E-01	2.94E-01	1.76E+01	1.76E+01
	Membrane	5.27E+03	5.24E+03	3.16E+05	3.16E+05
	CC	1.34E-01	1.34E-01	7.96E+00	7.96E+00
**Operation**	Catholyte	3.69E+01	3.68E+01	1.19E+04	1.19E+04
	Anolyte	3.68E+01	3.69E+01	1.19E+04	1.19E+04
	MES Operation	1.01E+06	2.17E+05	1.01E+06	2.17E+05
	CO_2 capture	1.42E+05	3.05E+04	1.42E+05	3.05E+04
	CO_2 utilization	-1.47E+06	-1.47E+06	-1.47E+06	-1.47E+06
**Extraction**		1.21E+07	2.52E+06	1.21E+07	2.52E+06
**TOTAL**		**1.18E+07**	**1.30E+06**	**1.23E+07**	**1.81E+06**

Table S6: NREU (MJ) for producing 1000t/year of AA using MES as described in Scenario 1, 2, 3 and 4.

		NREU (MJ)			
		**Scenario 1**	**Scenario 2**	**Scenario 3**	**Scenario 4**
**Reactor**	Cathode	7.29E+04	7.29E+04	4.38E+06	4.38E+06
	Anode	6.97E+00	6.97E+00	4.18E+02	4.18E+02
	Membrane	2.09E+04	2.08E+04	1.26E+06	1.26E+06
	CC	1.87E+00	1.87E+00	1.11E+02	1.11E+02
**Operation**	Catholyte	3.85E+02	3.83E+02	1.24E+05	1.24E+05
	Anolyte	3.83E+02	3.85E+02	1.24E+05	1.24E+05
	MES Operation	2.61E+07	2.97E+06	2.61E+07	2.97E+06
	CO_2 capture	3.67E+06	4.18E+05	3.67E+06	4.18E+05
**Extraction**		1.92E+08	3.45E+07	1.92E+08	3.45E+07
**TOTAL**		**2.22E+08**	**3.80E+07**	**2.28E+08**	**4.37E+07**

References:

Minke, C., Kunz, U. and Turek, T., 2017. Carbon felt and carbon fiber-A techno-economic assessment of felt electrodes for redox flow battery applications. Journal of Power Sources, 342, 116-124.

Dey, R.S., Hjuler, H.A. and Chi, Q., 2015. Approaching the theoretical capacitance of graphene through copper foam integrated three-dimensional graphene networks. Journal of Materials Chemistry A, 3(12), 6324-6329.

Evangelisti, S., Tagliaferri, C., Brett, D.J. and Lettieri, P., 2017. Life cycle assessment of a polymer electrolyte membrane fuel cell system for passenger vehicles. Journal of cleaner production, 142, 4339-4355.
